# Mixed pain: clinical practice recommendations

**DOI:** 10.3389/fmed.2025.1659490

**Published:** 2025-10-09

**Authors:** Giustino Varrassi, Giacomo Farì, Marco Antonio Narvaez Tamayo, Maria Patricia Gomez, Aura Marixa Guerrero Liñeiro, Carla Leal Pereira, Ezzat Samy Aziz, Christopher Gharibo, Alan D. Kaye, Luis Garcia-Larrea, Eleni Moka, Andrzej Król, Thomas Volk, Ameen A. Al-Alwany, Matteo Luigi Giuseppe Leoni

**Affiliations:** ^1^Fondazione Paolo Procacci (FPP), Rome, Italy; ^2^Department of Experimental Medicine, University of Salento, Lecce, Italy; ^3^Federación Latinoamericana de Asociaciones para el Estudio del Dolor (FEDELAT), La Paz, Bolivia; ^4^Pain Management Unit, Hospital Obrero, La Paz, Bolivia; ^5^Department of Anesthesia, University of Colombia, Bogotá, Colombia; ^6^Colsubsidio Health Research Center, Bogotá, Colombia; ^7^Pain Center, São Luis Hospital, São Paulo, Brazil; ^8^African Society of Regional Anaesthesia (AFSRA), Cairo, Egypt; ^9^Department of Anesthesia, Cairo University, Cairo, Egypt; ^10^Pain Management Center, New York University, New York, NY, United States; ^11^Department of Anesthesia, Louisiana State University Health Shreveport, Shreveport, LA, United States; ^12^University Claude Bernard Lyon 1, Lyon Center for Neuroscisnce-INSERM U1028, Lyon, France; ^13^European Society of Regional Anaesthesia and Pain Therapy (ESRA), Heidelberg, Germany; ^14^Anaesthesiology Department, Creta Interclinic Hospital, Hellenic Healthcare Group (HHG), Heraklion, Greece; ^15^Department of Anaesthesia and Chronic Pain Service, St George’s University Hospital, London, United Kingdom; ^16^Department of Anaesthesia, Intensive Care and Pain Therapy, Saarland University Medical Center and Saarland University Faculty of Medicine, Homburg, Germany; ^17^College of Medicine, University of Baghdad, Baghdad, Iraq; ^18^Department of Medical and Surgical Sciences and Translational Medicine, Sapienza University of Roma, Rome, Italy

**Keywords:** mixed pain, multimodal analgesia, neuropathic pain, nociceptive pain, nociplastic pain, interdisciplinary care, pain management, pain

## Abstract

Mixed pain, defined by the concurrent involvement of nociceptive, neuropathic, and sometimes nociplastic mechanisms, poses a significant diagnostic and therapeutic challenge within modern pain medicine. This complex pain phenotype is increasingly recognized as a prevalent and burdensome clinical entity, yet it remains substantially underdiagnosed and sub-optimally managed across diverse healthcare settings. Epidemiological data indicate that mixed pain affects a substantial proportion of patients with chronic pain syndromes and is consistently associated with more severe symptomatology, prolonged pain duration, functional impairment, diminished quality of life, and escalated healthcare resource utilization compared to pain of a single mechanism. In response to this unmet clinical need, the present recommendations aim to provide a structured, evidence-informed framework for the diagnosis and management of mixed pain. Developed through a rigorous process involving systematic literature review and multidisciplinary expert consensus, this document emphasizes the importance of mechanism-based therapeutic strategies tailored to the individual patient’s pain profile. Central to the approach is the implementation of multimodal and interdisciplinary care models that address the biological, psychological, and functional dimensions of mixed pain. These recommendations are intended for a broad spectrum of healthcare professionals, including primary care physicians, pain specialists, neurologists, oncologists, physiatrists, nurses, pharmacists, physical and occupational therapists, and clinical psychologists. The target population encompasses patients affected by mixed pain conditions such as chronic low back pain with radiculopathy, cancer-related pain, persistent post-surgical pain, and osteoarthritis complicated by central sensitization. By facilitating accurate diagnosis and integrated treatment planning, these recommendations seek to advance clinical practice, reduce the burden of mixed pain, and enhance patient-centered outcomes. This guidance aims to transform mixed pain care by promoting mechanism-based, multidisciplinary strategies with direct clinical applicability.

## 1 Introduction

Mixed pain represents a complex clinical phenomenon, defined by the concurrent presence of nociceptive and neuropathic pain mechanisms within a single pain condition (1). Nociceptive pain arises from tissue injury or inflammation with activation of nociceptors and microglia (2), while neuropathic pain results from lesions or diseases of the somatosensory nervous system (3). Additionally, nociplastic pain (a mechanism involving altered nociceptive processing without clear evidence of tissue or nerve damage) has been identified as a further contributor in mixed pain states (4, 5). The coexistence of these mechanisms leads to a heterogeneous symptom profile, often blending dull, aching sensations with burning or electric shock-like features.

Mixed pain is prevalent across numerous acute and chronic conditions, such as low back pain with nerve root involvement or spinal stenosis (6), cancer pain associated with tumor invasion and nerve compression (7), post-surgical pain syndromes (e.g., post-mastectomy, post-thoracotomy) (8, 9), and musculoskeletal diseases with central sensitization (e.g., osteoarthritis, rheumatoid arthritis) (10, 11). However, despite its high prevalence, mixed pain remains frequently underdiagnosed, contributing to suboptimal treatment outcomes (12). This diagnostic challenge reflects the overlap of nociceptive and neuropathic features, often obscuring the underlying mechanisms. Screening tools such as the painDETECT questionnaire facilitate recognition of neuropathic components and aid in the identification of mixed pain, with validation for adult (13) and pediatric (14) populations.

Management of mixed pain is inherently challenging due to its mechanistic heterogeneity, variable clinical presentation, and partial responsiveness to unimodal treatments (1, 12–15). NSAIDs may alleviate inflammation-driven nociceptive pain, while having limited efficacy for neuropathic components (16); conversely, agents such as gabapentinoids or antidepressants target neuropathic mechanisms but may not address nociceptive or inflammatory pain (17). Therefore, multimodal and mechanism-based therapeutic strategies, combining pharmacologic interventions (NSAIDs, opioids, gabapentinoids, antidepressants) with non-pharmacologic modalities (physical rehabilitation, psychological support, neuromodulation and interventional procedures), are recommended.

Emerging evidence underscores the role of central sensitization in mixed pain; wherein prolonged nociceptive input induces alterations in central pain processing (18, 19). This supports treatment approaches that address both peripheral and central pain pathways. The IASP definition of pain captures its multidimensional nature, with mixed pain providing a clear clinical example (20). Recent IASP and ICD-11 classifications promote improved categorization of mixed pain, facilitating accurate diagnosis and individualized treatment planning (21). Given the multidimensional nature of pain, the biopsychosocial model provides an essential framework for understanding mixed pain. This model recognizes that biological drivers (e.g., nociceptive, neuropathic, and nociplastic mechanisms) interact with psychological processes (e.g., mood, coping strategies, catastrophizing) and social factors (e.g., work, family, cultural expectations) to shape the pain experience and influence outcomes. Mixed pain is associated with greater intensity, longer duration, impaired quality of life, increased healthcare utilization, and higher disability rates relative to pure nociceptive or neuropathic pain states (22). In this review, “multimodal” refers to the coordinated use of pharmacological, interventional, physical, and psychological therapies to achieve synergistic pain relief while minimizing reliance on any single treatment, whereas “mechanism-based” denotes tailoring interventions to the underlying drivers of mixed pain—nociceptive, neuropathic, or nociplastic—by selecting therapies such as anti-inflammatories, gabapentinoids, or cognitive-behavioral strategies that specifically target these mechanisms. The present recommendations aim to provide an evidence-based, clinically relevant framework for the diagnosis and management of mixed pain, supporting healthcare providers across disciplines in delivering effective, patient-centered care.

### 1.1 Scope and target audience

The main aim of the research was to provide a structured, evidence-based framework for the diagnosis and management of mixed pain. The guidance is organized around key clinical questions developed using the PICO (Population, Intervention, Comparator, Outcomes) methodology. It addresses: (1) diagnostic strategies, comparing validated screening tools combined with clinical assessment versus clinical assessment alone; (2) pharmacological management, comparing multimodal pharmacotherapy to monotherapy; and (3) interdisciplinary care, contrasting integrated pain management programs with single-discipline approaches. Outcomes of interest include diagnostic accuracy, treatment appropriateness, pain relief, functional improvement, quality of life, and healthcare resource utilization.

The document is intended for implementation across diverse healthcare settings (primary care, pain specialty clinics, hospitals, rehabilitation centers, and multidisciplinary programs) by a broad range of clinicians, including physicians, advanced practice providers, pharmacists, rehabilitation therapists, and mental health professionals. The framework promotes adaptable, patient-centered, and high-quality care.

## 2 Methods

These clinical practice recommendations were developed using a modified consensus approach, recognizing the limited availability of high-quality randomized controlled trials specifically addressing mixed pain management. The development process integrated best available evidence with expert clinical consensus and real-world practice considerations. The following scientific societies were involved in the development of these clinical recommendations:

- Fondazione Paolo Procacci (FPP)
- African Society of Regional Anesthesia (AFSRA)
- European Society of Regional Anaesthesia & Pain Therapy (ESRA)
- Federación Latinoamericana de Asociaciones para el Estudio del Dolor (FEDELAT)

### 2.1 Development group and process

A multidisciplinary expert panel was assembled, including different specialists, including methodological experts. The panel acknowledged that traditional guideline methods based on high-quality comparative trials were unfeasible due to limited mixed pain research. A pragmatic evidence review was adopted. Patient representatives were engaged during the initial project design to provide perspectives from individuals with lived experience of mixed pain. However, consensus could not be formally established, primarily due to the highly technical nature of the methodological processes involved in developing these recommendations. We emphasize that future initiatives should incorporate structured patient involvement, particularly in the prioritization of research questions and in the translation of recommendations into practical clinical pathways.

These clinical practice recommendations address the diagnosis and management of mixed pain through a systematic approach based on clearly defined clinical questions formulated using the PICO (Population, Intervention, Comparator, Outcomes) framework. The first clinical question examines diagnostic assessment approaches in adults with suspected mixed pain conditions, comparing validated screening tools (DN4, PainDETECT, LANSS) plus comprehensive clinical assessment against clinical assessment alone, with outcomes focusing on diagnostic accuracy, appropriate treatment selection, and time to diagnosis. The second question evaluates pharmacological management by comparing multimodal pharmacotherapy combinations against monotherapy approaches in adults with diagnosed mixed pain, measuring outcomes of pain reduction (≥30% improvement), functional improvement, quality of life scores, and adverse events. The third question investigates interdisciplinary care approaches, comparing comprehensive pain management programs with single-discipline care approaches in adults with chronic mixed pain, evaluating outcomes including pain intensity scores, functional status measures, healthcare utilization, and patient satisfaction.

Literature search strategy: A systemic comprehensive search (1990–2024) was performed in MEDLINE/PubMed, Cochrane CENTRAL, Embase, CINAHL, PsycINFO, and Web of Science, using terms such as “mixed pain,” “neuropathic pain,” “nociceptive pain,” “central sensitization,” “multimodal analgesia,” “pain assessment,” “pain management” and “chronic pain.”

### 2.2 Evidence inclusion

Given the limited clinical trials on mixed pain as a distinct entity, the evidence base was broadened to include RCTs in relevant populations, high-quality observational studies, systematic reviews, and meta-analyses on multimodal strategies. Where direct evidence was lacking, expert consensus, position statements, and real-world clinical data informed the recommendations.

### 2.3 Evidence quality assessment

Given the previously mentioned scarcity of trials, a modified framework was used to assess evidence quality in place of the traditional GRADE method. This approach prioritized study design, methodological rigor, applicability to mixed pain, consistency of outcomes, clinical relevance, safety, and feasibility. Expert consensus supplemented gaps in empirical data to ensure evidence-informed, practical recommendations. Due to the limited presence of articles specifically addressing mixed pain, GRADE was not feasible, as it is normally applied to bodies of evidence dominated by RCTs. To address gaps where direct evidence was lacking, we integrated a structured expert consensus (modified Delphi).

### 2.4 Recommendation development process

#### 2.4.1 Consensus method

Recommendations were developed through structured expert consensus using a modified Delphi approach, recognizing that traditional evidence-based recommendations were not feasible given the current state of research. The complete methodology used for consensus development and results of the voting process are reported as Supplementary material.

#### 2.4.2 Recommendation categories

Given the absence of RCTs specific to mixed pain, recommendation strength was determined by integrating expert consensus levels from our Delphi process with available clinical evidence:

Strong recommendations: Assigned when ALL of the following criteria were met:

- Expert consensus ≥85% in the Delphi process (achieved by 11 of our 16 statements)
- Consistent evidence from observational studies or established efficacy in component pain mechanisms (nociceptive and/or neuropathic)
- Favorable benefit-risk profile documented in real-world clinical practice
- Feasibility of implementation across diverse healthcare settings

Conditional recommendations: Assigned when any of the following applied:

- Expert consensus 70%–84% in the Delphi process (achieved by 5 of our 16 statements)
- Limited to extrapolated evidence from single pain mechanisms
- Moderate consensus despite theoretical benefit (as seen with imaging/electrophysiology, dual-mechanism opioids, and topical agents, all achieving 80% consensus)
- Variable benefit-risk ratios across patient subgroups or healthcare settings

Evidence levels were assigned as follows: High evidence required systematic reviews demonstrating effectiveness in both nociceptive and neuropathic pain populations, large registry data (>1,000 patients) with mixed pain phenotypes, or established guidelines with >10 years implementation; Moderate evidence required strong evidence in either pain mechanism with biological plausibility for mixed pain, or multiple observational studies (≥3) in heterogeneous pain populations; Low evidence included extrapolated data from related conditions, expert consensus, or limited observational studies; Very low evidence was restricted to case series or theoretical frameworks.

#### 2.4.3 Limitations and transparency

The panel acknowledges key limitations: limited RCTs specifically addressing mixed pain necessitated reliance on indirect evidence and expert opinion; heterogeneity of mixed pain complicates universal recommendations; the evolving nature of mixed pain research may impact future guidance; and practical implementation may vary across healthcare systems, requiring local adaptation.

## 3 Results

### 3.1 Pathophysiology and mechanisms

Understanding the pathophysiology of mixed pain is essential for accurate diagnosis and personalized treatment. Mixed pain syndromes are characterized by the concurrent activation of nociceptive (2, 23) and neuropathic (3, 24) pathways, which may operate independently or interact synergistically at peripheral and central levels. This overlap enhances nociceptive transmission and complicates both pharmacological and interventional management (25). Recognizing these mechanisms is critical for optimizing multimodal pain strategies.

#### 3.1.1 Nociceptive component

The nociceptive component arises from inflammation or tissue injury, leading to activation of peripheral nociceptors (23). Pain signals transmitted via Aδ and C fibers typically produce localized, aching, or throbbing sensations (26), reflecting a physiological response to noxious stimuli. This mechanism predominates in conditions such as osteoarthritis and post-surgical pain.

#### 3.1.2 Neuropathic component

Neuropathic pain results from direct injury or disease of the peripheral or central somatosensory system (27). Patients commonly describe burning, electric, shooting, or tingling sensations (24), reflecting aberrant neuronal excitability. Sensory disturbances can manifest as both “negative” symptoms (hypoesthesia, numbness) or “positive” symptoms, such as allodynia and hyperalgesia, which indicate altered pain modulation at spinal and supraspinal levels (28). Identifying these features is essential for accurate diagnosis and guiding multimodal therapy. These parameters were selected for the clinical recommendations because they represent core descriptors consistently reported in neuropathic pain populations, are embedded within the IASP NeuPSIG diagnostic criteria, and serve as reproducible markers in both clinical and research contexts. Their inclusion was therefore guided by their high clinical relevance and their utility in distinguishing neuropathic features from other pain mechanisms.

#### 3.1.3 Nociplastic pain

Nociplastic pain, defined as arising from altered nociceptive processing without evident tissue or nerve injury, increasingly contributes to mixed pain states (29, 30). Frequently observed in fibromyalgia and chronic low back pain, it amplifies pain perception and complicates treatment. Despite some criticism of this concept (31), its recognition enhances the understanding and management of complex pain syndromes.

#### 3.1.4 Central sensitization

Central sensitization, marked by heightened responsiveness of nociceptive neurons in the central nervous system (32), leads to amplified pain signals, increased intensity, pain spreading beyond the initial site, and persistence after tissue healing. Mechanisms include increased synaptic efficacy, reduced inhibition, and glial activation (33, 34). In chronic low back pain, osteoarthritis with sensitization, and post-surgical pain, central sensitization sustains and intensifies symptoms. Clinical signs include hyperalgesia and allodynia, which complicate management, necessitating multimodal approaches (35).

#### 3.1.5 Examples of mixed pain conditions

As already explained, mixed pain involves nociceptive, neuropathic, nociplastic components, and central sensitization. Representative conditions include:

##### 3.1.5.1 Low back pain with radiculopathy

Combines nociceptive musculoskeletal pain with neuropathic elements from nerve root compression (36), manifesting as localized back pain with radiating leg symptoms.

##### 3.1.5.2 Cancer-related pain

Arises from tumor-induced tissue damage (nociceptive) and treatment-related neuropathy (e.g., chemotherapy-induced peripheral neuropathy - CIPN) (37, 38), necessitating multimodal management.

##### 3.1.5.3 Post-surgical pain

Results from tissues and nerve trauma during surgery and subsequent inflammation (39), contributing to chronic post-surgical pain syndromes that require comprehensive treatment (40, 41).

##### 3.1.5.4 Osteoarthritis with central sensitization

While traditionally nociceptive, OA pain may involve central sensitization, heightening pain disproportionate to joint damage (42). Recognizing these mixed mechanisms is critical for effective intervention targeting multiple pain pathways.

**Recommendation 1**

**Mechanism-based classification.** We strongly recommend that clinicians classify mixed pain based on the relative contributions—i.e., the estimated proportion of the overall pain experience—attributable to nociceptive, neuropathic, and nociplastic mechanisms, as determined by patient-reported symptoms, clinical examination findings, and validated screening tools, in order to guide targeted therapeutic approaches (Strong recommendation, Low evidence).

**Recommendation 2**

**Central sensitization assessment.** We suggest routine evaluation for clinical signs of central sensitization (hyperalgesia, allodynia, temporal summation) in patients with suspected mixed pain to inform treatment planning (Conditional recommendation, Low evidence).

### 3.2 Diagnostic approach

An accurate diagnostic approach to mixed pain requires systematic use of evidence-based assessment methods to identify the presence and relative contribution of different pain mechanisms. Based on a comprehensive evidence review, we present structured recommendations for diagnostic assessment, combining validated screening tools with clinical evaluation to optimize diagnostic accuracy and guide treatment selection.

#### 3.2.1 Clinical assessment

##### 3.2.1.1 Pain history

A thorough pain history should document onset, duration, intensity, temporal pattern, aggravating and relieving factors. Qualitative descriptors (e.g., stabbing, burning, aching) and associated symptoms such as numbness or weakness are critical for differential diagnosis and management.

##### 3.2.1.2 Physical examination

Physical examination should include inspection, palpation, range of motion testing, and sensory evaluation (light touch, pinprick, thermal perception). Assessment of motor strength and deep tendon reflexes aids in detecting neurological or musculoskeletal impairments.

#### 3.2.2 Screening tools

Screening tools are helpful for identifying neuropathic components within mixed pain. DN4 is a clinician-administered tool with high sensitivity and specificity for neuropathic pain (43). PainDETECT is a validated self-reported questionnaire for chronic back pain and other conditions (44). LANSS integrates patient-reported symptoms with bedside sensory testing, facilitating early diagnosis (45). These tools are widely endorsed for clinical use.

#### 3.2.3 Imaging and electrophysiology

Imaging and electrophysiological studies help elucidate underlying causes. MRI effectively identifies structural abnormalities (disc herniation, spinal stenosis, nerve root compression) (46). EMG and NCS assess peripheral nerve function in radiculopathy, neuropathies, and plexopathies (47). Bone scintigraphy identifies inflammatory or neoplastic lesions; musculoskeletal ultrasound dynamically evaluates soft tissue structures without radiation and is recommended for diagnosing musculoskeletal conditions and assessing peripheral nerves allowing for detection of nerve entrapment, inflammation, and structural abnormalities (48, 49). However, imaging findings often lack correlation with clinical symptoms, particularly for facet joint or sacroiliac (SI) joint pain, which together account for over half of chronic spinal pain cases. Studies consistently show degenerative changes on MRI do not reliably predict facet joint pain (50).

Similarly, imaging for SI joint pain demonstrates limited diagnostic accuracy (CT: sensitivity 57.5%, specificity 69%; bone scans: sensitivity 100%, specificity 12.9%) (51). Degenerative findings on spinal imaging are also common in asymptomatic individuals, complicating interpretation (52).

#### 3.2.4 Red and yellow flags

Red flags indicate serious pathology (infection, malignancy, neurological compromise) requiring urgent management (53). Yellow flags refer to psychosocial risk factors (depression, fear-avoidance, catastrophizing) that predict chronicity and poor outcomes, particularly in adolescents (54, 55).

#### 3.2.5 Diagnosis by exclusion

A diagnosis of exclusion requires systematically ruling out referred pain, functional pain syndromes (e.g., fibromyalgia), and somatization disorders to ensure appropriate management (56, 57).

**FIGURE 1:**
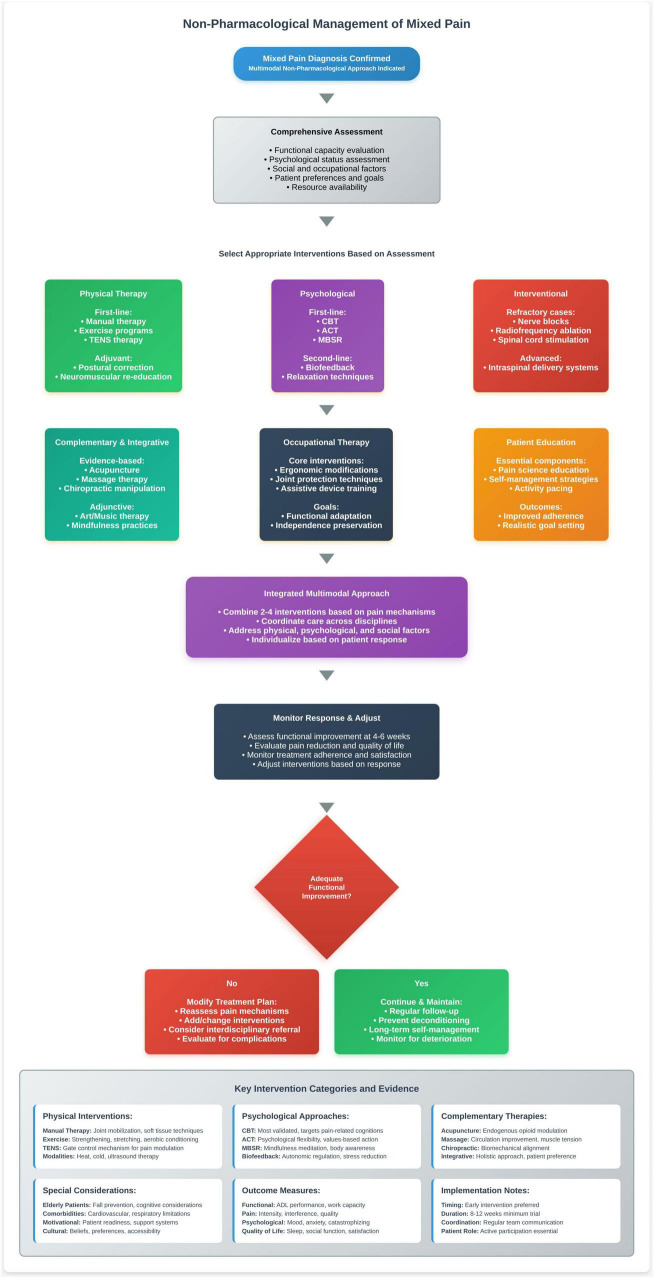
Diagnostic algorithm for the evaluation of mixed pain. The flowchart presents a structured approach beginning with comprehensive clinical assessment (pain history and physical examination), followed by application of validated screening tools (DN4, PainDETECT, LANSS) to identify neuropathic components. Based on screening results, patients undergo targeted investigations including advanced imaging (MRI), electrophysiological studies (EMG/NCS), or musculoskeletal-focused assessments. The diagnostic pathway culminates in classification as pure nociceptive, pure neuropathic, or mixed pain, enabling mechanism-based treatment selection. Key decision points and diagnostic modalities are highlighted to guide clinical practice.

Figure 1 presents a diagnostic algorithm differentiating mixed pain from pure nociceptive or neuropathic pain syndromes, integrating clinical assessment, validated screening tools, and targeted investigations.

**Recommendation 3:**

**Use of validated screening tools:** We strongly recommend that clinicians use validated screening tools, specifically the DN4 (Douleur Neuropathique en 4 Questions), PainDETECT, or LANSS (Leeds Assessment of Neuropathic Symptoms and Signs), in combination with comprehensive clinical assessment to identify neuropathic components in patients with suspected mixed pain (Strong recommendation, Moderate evidence).

**Recommendation 4:**

**Comprehensive clinical assessment:** We strongly recommend that diagnostic evaluation include a comprehensive clinical assessment, encompassing detailed pain history, physical examination with sensory testing, functional status, and systematic appraisal of psychosocial factors (e.g., emotional distress, fear-avoidance, catastrophizing, and social support). This biopsychosocial approach enables more accurate diagnosis and guides mechanism-based, patient-centered management strategies (Strong recommendation, Low evidence).

**Recommendation 5:**

**Imaging and electrophysiology** We suggest using advanced imaging (MRI) and electrophysiological studies (EMG/NCS) selectively based on clinical presentation and screening tool results rather than routinely in all mixed pain patients (Conditional recommendation, Low evidence).

**Recommendation 6:**

**Red and yellow flag assessment** We strongly recommend systematic screening for red flags (serious pathology) and yellow flags (psychosocial risk factors) during initial evaluation of all mixed pain patients (Strong recommendation, Moderate evidence)

### 3.3 Pharmacological management

Pharmacological management of mixed pain requires a paradigm shift from single-mechanism approaches toward evidence-based multimodal strategies targeting nociceptive, neuropathic, and nociplastic components. Treatment must be mechanism-based and multimodal (12, 58), as monotherapy rarely suffices. Combination therapies targeting multiple pain pathways often yield superior analgesia with acceptable safety profiles when appropriately implemented (59).

#### 3.3.1 General principles

Management should be individualized, considering clinical phenotype, comorbidities, and drug tolerability. A “start low, go slow” approach minimizes adverse effects, especially in older adults or polypharmacy patients (60). Oral formulations are preferred for ease of use and safety, unless rapid or regional delivery is required (61). Regular reassessment is critical for optimizing efficacy and ensuring safety (62).

#### 3.3.2 Drug classes

##### 3.3.2.1 Non-steroidal anti-inflammatory drugs (NSAIDs)

Non-steroidal anti-inflammatory drugs inhibit cyclooxygenase (COX) enzymes, reducing prostaglandin synthesis and inflammatory nociceptive signaling (63). They effectively target inflammatory pain in musculoskeletal and arthritic conditions. Common agents include ibuprofen (64), ibuprofen arginine (41), naproxen (65), diclofenac (66), ketoprofen (67), dexketoprofen (68), and celecoxib (69). All carry gastrointestinal, renal, and cardiovascular risks, which increase with dose and duration.

##### 3.3.2.2 Acetaminophen (paracetamol)

Acetaminophen is effective for mild-to-moderate nociceptive pain and well tolerated in multimodal regimens through multiple mechanisms, including central COX inhibition, serotonergic modulation, action through cannabinoid channels, effects on TRPV1 channels, and the action of its metabolite AM404 (70).

##### 3.3.2.3 Opioids

Opioids provide effective analgesia for moderate-to-severe mixed pain, acting via μ-opioid receptor agonism on spinal and supraspinal pathways. They also influence neuropathic pathways, proving useful in cancer, postoperative, and refractory chronic mixed pain (61). Common agents include morphine, oxycodone, buprenorphine, tramadol, and tapentadol. Tramadol and tapentadol combine opioid action with monoaminergic reuptake inhibition, enhancing efficacy for neuropathic pain (71, 72). Risks include tolerance, dependence, hyperalgesia, and misuse, necessitating careful monitoring (73). Of note, opioids have immunosuppressive effects, including natural killer (NK) cell inhibition, raising infection risks and potentially promoting tumor progression (74).

##### 3.3.2.4 Anticonvulsants

Gabapentin and pregabalin modulate voltage-gated calcium channels via α2δ subunit binding, reducing excitatory neurotransmission in neuropathic pathways (75). They alleviate symptoms such as burning and electric pain (61), and are used in post-surgical, cancer-related, and musculoskeletal mixed pain. Side effects include sedation, dizziness, and edema, requiring cautious titration (28).

##### 3.3.2.5 Antidepressants

Serotonin-norepinephrine reuptake inhibitors (SNRIs) (e.g., duloxetine) and tricyclic antidepressants (TCAs) (e.g., amitriptyline, nortriptyline) inhibit serotonin and norepinephrine reuptake, enhancing descending inhibitory pathways (61). Duloxetine is effective in chronic low back pain; TCAs are used for neuropathic syndromes (76). TCAs pose anticholinergic and cardiac risks, particularly in elderly patients, requiring ECG monitoring (77).

##### 3.3.2.6 Topical agents

Topical agents target localized neuropathic pain. Lidocaine patches block sodium channels, reducing ectopic discharges (60). Capsaicin creams and patches desensitize TRPV1 receptors, reducing nociceptor activity (78). These options are advantageous in elderly or polypharmacy patients due to minimal systemic effects (79).

##### 3.3.2.7 NMDA receptor antagonists

NMDA antagonists are useful for refractory mixed pain with central sensitization. Low-dose ketamine infusions reduce central hyperexcitability in post-surgical or cancer pain (80). Dextromethorphan has potential for neuropathic pain but is limited by psychomimetic effects and variable efficacy (81). These agents are reserved for specialized care (82).

##### 3.3.2.8 Cannabinoids

Cannabinoids demonstrate therapeutic potential for mixed pain conditions, particularly those involving neuropathic and cancer-related components. Clinical evidence reveals variable analgesic efficacy, with studies showing modest pain relief benefits alongside improvements in sleep quality and functional outcomes (83). However, significant safety considerations must be weighed, including risks of cognitive dysfunction, psychological disturbances, and potential for cannabis use disorder, particularly with chronic THC exposure (84, 85). The endocannabinoid system’s interaction with both nociceptive and neuropathic pain pathways through CB1 and CB2 receptor modulation provides a mechanistic rationale for multimodal pain management, though optimal dosing protocols and long-term safety profiles remain incompletely characterized (86). Cannabis-based therapies should be considered within individualized treatment algorithms, with careful patient selection, regular monitoring for adverse effects and dependency, and implementation only within appropriate legal frameworks where permitted.

#### 3.3.3 Rational polypharmacy

Rational polypharmacy is central to mixed pain management. Combining agents with complementary actions (e.g., opioids with anticonvulsants, NSAIDs with antidepressants) enhances analgesia while minimizing individual drug doses and side effects (87, 88). Topical plus systemic therapy offers targeted pain control with fewer systemic risks, especially valuable in elderly or complex patients (89). This aligns with guidelines advocating personalized, mechanism-based care for complex pain syndromes (61).

#### 3.3.4 Fixed-dose combinations (FDCs)

**FIGURE 2:**
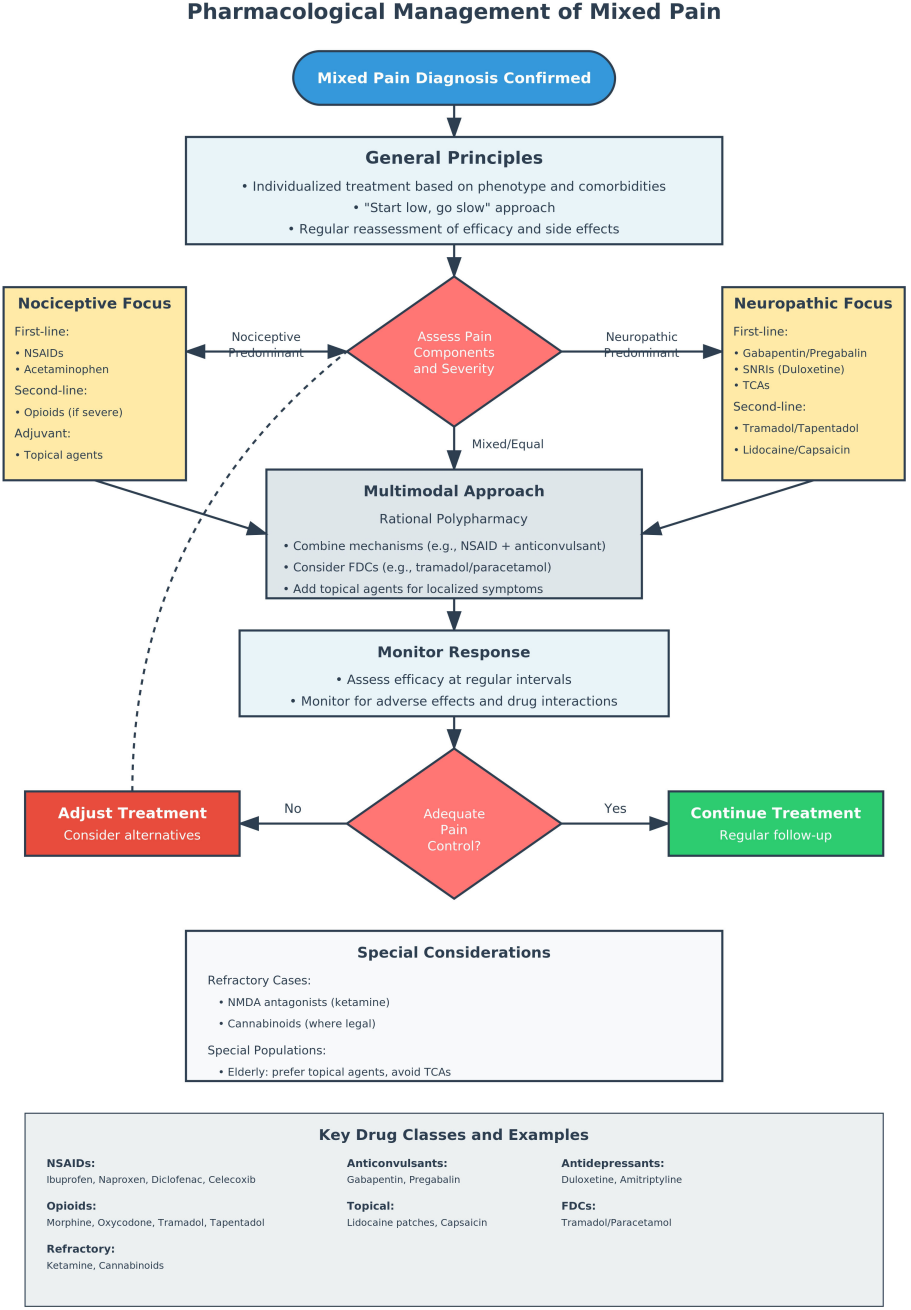
Pharmacological management algorithm for mixed pain.

Fixed-dose combinations (FDCs) offer practical, evidence-based options for mixed pain, particularly in musculoskeletal disorders (68, 90). Combinations such as tramadol/paracetamol deliver synergistic analgesia (μ-opioid modulation with prostaglandin inhibition) at lower doses with favorable safety (91). FDCs improve adherence, reduce pill burden, and minimize dosing errors (92), supporting their inclusion in multimodal pain strategies. Figure 2 illustrates the decision-making process for implementing evidence-based pharmacologic interventions in mixed pain, integrating the principles outlined in Recommendations 7–11.

First-line pharmacological combinations for mixed pain and their contraindications are resumed in Table 1.

**TABLE 1:** First-line pharmacological combinations for mixed pain and their contraindications.

Pain type	Recommended first-line combination	Dosage notes
Nociceptive-dominant mixed pain	• Ibuprofen 400 mg TID + Gabapentin 300 mg TID, titrate to 1800 mg/day • Acetaminophen 1 g TID + Duloxetine 30 mg daily, increase to 60 mg	Titrate gabapentin gradually to improve tolerability. Monitor for renal function with NSAIDs.
Neuropathic-dominant mixed pain	• Pregabalin 75 mg BID, titrate to 300 mg BID + Topical lidocaine 5% • Duloxetine 30–60 mg daily + Tramadol 50 mg QID, max 400 mg/day	Use caution with tramadol in elderly and serotonergic drugs. Titrate pregabalin slowly.

**Recommendation 7:**

**Multimodal pharmacotherapy approach** We strongly recommend multimodal pharmacotherapy combining agents with different mechanisms of action rather than monotherapy for mixed pain management (Strong recommendation, Moderate evidence).

**Recommendation 8**:

**First-line Combination Therapy**

We suggest combining NSAIDs or acetaminophen with gabapentinoids (gabapentin or pregabalin) or serotonin-norepinephrine reuptake inhibitors (SNRIs) such as duloxetine as first-line therapy for most mixed pain conditions (Conditional recommendation, Low evidence).

**Recommendation 9**:

We suggest considering opioids with dual mechanisms of action, specifically tramadol or tapentadol, over traditional opioids when opioid therapy is indicated for mixed pain management (Conditional recommendation, Low evidence).

**Recommendation 10**:

**Topical agent** We suggest prioritizing topical agents (lidocaine patches, capsaicin) for localized mixed pain, particularly in elderly patients or those with multiple comorbidities (Conditional recommendation, Moderate evidence).

### 3.4 Non-pharmacological interventions

Pharmacological treatments alone are often inadequate for mixed pain management. Evidence supports multimodal approaches integrating non-pharmacological therapies—such as physical rehabilitation, psychological interventions (e.g., cognitive-behavioral therapy), and complementary modalities (e.g., art or music therapy)—to address nociceptive, neuropathic, and psychosocial components (40, 93, 94). These strategies address multiple pain dimensions, enhancing function, emotional well-being, and adherence (95). Multimodal management shows superior efficacy over pharmacotherapy alone in reducing pain and improving quality of life in acute and chronic pain (96, 97), and is now emphasized in guidelines for personalized, mechanism-based care.

#### 3.4.1 Physical therapy

Physical therapy is a key component of multimodal mixed pain management, targeting nociceptive and neuropathic mechanisms to reduce pain, restore function, and prevent deconditioning. Techniques include manual therapy, stretching, strengthening, postural correction, and neuromuscular re-education (98), particularly effective in chronic low back pain, postoperative pain, and fibromyalgia (99). Additionally, Transcutaneous Electrical Nerve Stimulation (TENS) serves as an adjunct by stimulating large-diameter afferent fibers and modulating central pain transmission via gate-control mechanisms (100). TENS is particularly valuable for patients unsuitable for systemic pharmacotherapy due to comorbidities or polypharmacy.

#### 3.4.2 Psychological interventions

Psychological interventions are essential in managing mixed pain, particularly chronic cases where cognitive, emotional, and behavioral factors exacerbate symptoms. In the context of mixed pain, maladaptive coping patterns include behaviors and cognitions such as pain catastrophizing (exaggerated negative orientation toward pain stimuli), fear-avoidance (avoiding movement or activity due to fear of worsening pain), low self-efficacy, and passive coping strategies (e.g., over-reliance on medications without active self-management). These patterns are consistently associated with greater pain intensity, disability, psychological distress, and poorer treatment outcomes. Conversely, adaptive strategies such as active coping, acceptance, and problem-focused behaviors are linked to better adjustment and functional improvement. Recognizing maladaptive coping early and addressing them through interventions such as cognitive-behavioral therapy (CBT), acceptance and commitment therapy (ACT), and mindfulness-based interventions is therefore critical to optimize long-term outcomes. CBT effectively reduces pain intensity, improves function, and mitigates fear-avoidance (101). ACT enhances psychological flexibility and coping (102), while Mindfulness-Based Stress Reduction (MBSR) alleviates distress and improves emotional regulation (103). These approaches are especially indicated for patients with high distress, fear-avoidance, or comorbid depression and anxiety, which amplify both nociceptive and neuropathic components in mixed pain syndromes.

#### 3.4.3 Interventional pain management

Interventional pain management is pivotal in mixed pain treatment, particularly for refractory cases or as adjunctive therapy. Nerve blocks (e.g., epidural steroids, peripheral nerve blocks) provide targeted relief of nociceptive and neuropathic components (104). Radiofrequency ablation of facet joints or dorsal root ganglia offers sustained reduction in segmental pain transmission, notably in spinal syndromes (105). Neuromodulation, such as spinal cord stimulation or non-invasive cortical stimulation (rTMS) benefits complex mixed pain, including persistent spinal pain syndrome and diabetic neuropathy (106). In advanced cancer pain, intraspinal drug delivery enables potent analgesia with fewer systemic effects (107). These techniques are most effective when integrated within a comprehensive, multidisciplinary treatment strategy tailored to the pain mechanisms involved. Nevertheless, the evidence base for interventional therapies in mixed pain syndromes remains limited, preventing their designation as standard care or their incorporation into evidence-based clinical recommendations.

#### 3.4.4 Complementary and integrative medicine

Complementary and integrative medicine (CIM) provides effective adjunctive strategies for managing mixed pain by targeting nociceptive and neuropathic components through non-pharmacological means. Acupuncture modulates endogenous opioids and central sensitization, reducing pain in chronic low back pain and neuropathy (108). Massage therapy alleviates musculoskeletal pain, improves circulation, and reduces muscle tension (109). Chiropractic manipulation, particularly spinal adjustments, addresses mechanical nociceptive pain and enhances biomechanical alignment (110). Biofeedback and relaxation techniques reduce pain-related distress, improve autonomic regulation, and support coping in persistent pain (111). When incorporated into a multidisciplinary treatment plan, these modalities enhance clinical outcomes and may decrease dependence on pharmacologic therapies. However, the current evidence supporting complementary and integrative medicine in mixed pain remains limited. While modalities such as acupuncture, massage therapy, and mindfulness-based interventions show promise in improving pain and quality of life, these findings are often based on small-scale or heterogeneous studies with variable methodological quality. Large, well-designed randomized controlled trials focusing specifically on mixed pain are urgently needed to establish efficacy, clarify mechanisms of action, and determine how these therapies can be optimally integrated within multimodal, interdisciplinary care frameworks.

#### 3.4.5 Occupational therapy

Occupational therapy contributes to comprehensive mixed pain management by promoting function, independence, and engagement in daily activities. Interventions—ergonomics, joint protection, and assistive devices—reduce biomechanical stress and disability (112). Addressing physical and psychosocial aspects, it improves coping and life participation, particularly in chronic or musculoskeletal mixed pain (113).

#### 3.4.6 Patient education

**FIGURE 3:**
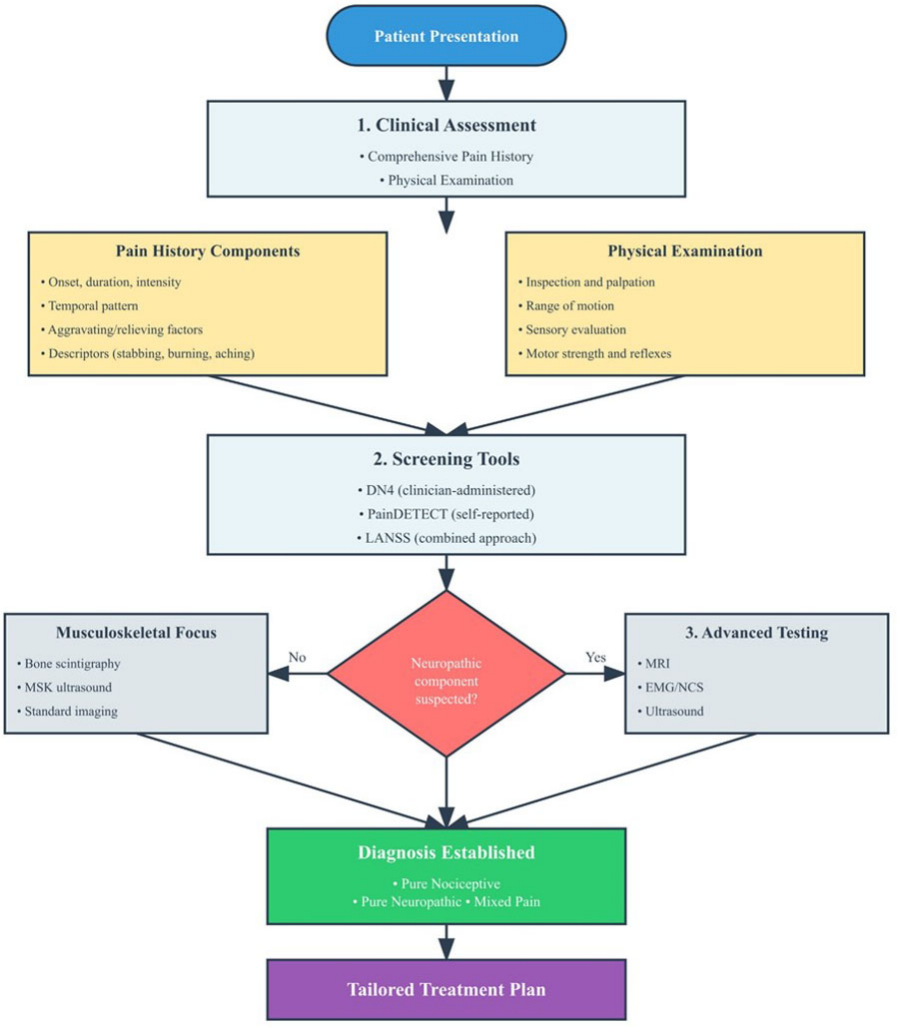
Non-pharmacological management algorithm for mixed pain. The flowchart presents a comprehensive decision-making framework for implementing non-pharmacological interventions in patients with confirmed mixed pain diagnosis. Following initial comprehensive assessment of functional capacity, psychological status, and patient-specific factors, the algorithm guides selection from six evidence-based intervention categories: physical therapy (manual therapy, exercise programs, TENS), psychological interventions (CBT, ACT, MBSR), interventional procedures for refractory cases, complementary and integrative medicine approaches, occupational therapy, and patient education.

Patient education is essential in mixed pain management, fostering understanding of its multifactorial nature, setting realistic goals (e.g., pain reduction), and promoting self-management through activity pacing and lifestyle modifications. Education enhances adherence and supports patient-centered care (114). Figure 3 illustrates coordinated, multimodal non-pharmacological strategies for addressing mixed pain complexity.

**Recommendation 11:**

**Multimodal non-pharmacological approach** We strongly recommend adopting multimodal approaches that combine pharmacological, interventional, and rehabilitative therapies to optimize outcomes and minimize adverse effects in mixed pain management (Strong recommendation, High evidence).

**Recommendation 12:**

**Early psychological intervention and patient education** We suggest early integration of psychological interventions (e.g., CBT, ACT, MBSR) for patients with mixed pain, particularly those with high pain-related distress or maladaptive coping patterns (Conditional recommendation, Moderate evidence). We strongly recommend providing comprehensive patient education about the nature of mixed pain, realistic treatment goals, and self-management strategies as a fundamental component of care (Strong recommendation, Low evidence).

### 3.5 Interdisciplinary care and rehabilitation

Mixed pain requires a comprehensive approach due to its multifactorial biopsychosocial nature. Effective management involves coordinated input from physicians, psychologists, physiotherapists, and allied health professionals to optimize function and quality of life. Interdisciplinary care improves pain intensity, emotional well-being, and disability in complex pain presentations, underscoring its value in modern pain medicine (115, 116).

#### 3.5.1 Core team members

A comprehensive approach integrates diverse expertise to enhance biopsychosocial outcomes.

3.5.1.1 Physicians (general practitioners, pain specialists, neurologists, oncologists, rheumatologists) conduct clinical and neurophysiological assessments and coordinate mechanism-based therapies (117).

3.5.1.2 Nurses, including pain nurses and case managers, support care coordination, deliver education, and enhance adherence (118).

3.5.1.3 Pharmacists optimize polypharmacy management, reducing drug interactions and improving regimen safety (119).

3.5.1.4 Physiotherapists implement exercise and manual therapies to restore mobility and reduce sensitization (120).

3.5.1.5 Occupational Therapists promote independence through adaptive strategies and task modification (121).

3.5.1.6 Psychologists provide cognitive-behavioral and psychotherapeutic interventions to address distress and maladaptive coping (122).

3.5.1.7 Social Workers address socioeconomic barriers, connect patients to services, and advocate for resources (123).

Evidence shows that interdisciplinary programs involving at least three disciplines, with coordinated or virtual collaboration, yield superior outcomes in pain relief, function, and satisfaction compared to monodisciplinary care (124).

#### 3.5.2 Multidisciplinary vs. interdisciplinary

Interdisciplinary management surpasses multidisciplinary models. While multidisciplinary teams operate in parallel, interdisciplinary care employs integrated plans, shared decision-making, and unified goals. Evidence demonstrates that interdisciplinary multimodal pain treatment:

reduces pain and improves function and quality of life, with sustained benefits (125);

lowers healthcare utilization, decreasing reliance on specialty services and diagnostics (126);

increases return-to-work rates through integrated biopsychosocial programs (127).

These findings support interdisciplinary rehabilitation as superior for mitigating mixed pain burden.

#### 3.5.3 Rehabilitation strategies

Effective rehabilitation combines structured, evidence-based interventions:

- Goal setting with SMART (specific, measurable, achievable, relevant, time-bound) goals enhances engagement and tracks progress (128).
- Functional restoration prioritizes meaningful activity participation over complete pain elimination (129).
- Gradual activity pacing via quota-based models balances activity and rest, reducing boom-bust cycles and improving quality of life (130).
- Pain self-management programs, integrating education, activity, and psychological support, improve independence, mental health, and quality of life (131).

These strategies reinforce a biopsychosocial model for mixed pain, promoting resilience, patient agency, and functional recovery beyond symptom control.

**Recommendation 13:**

**Interdisciplinary team composition and regular team communication.** We strongly recommend that interdisciplinary teams for mixed pain include at minimum: physician, nurse, pharmacist, physiotherapist, and psychologist, with additional specialists as clinically indicated (Strong recommendation, High evidence). We suggest establishing regular interdisciplinary team meetings and communication protocols to ensure coordinated care and treatment plan optimization (Conditional recommendation, Low evidence).

### 3.6 Monitoring and outcome assessment

Long-term management of mixed pain requires ongoing, systematic evaluation of therapeutic efficacy and patient safety. Regular outcome monitoring ensures treatments remain aligned with SMART patient goals while facilitating timely therapeutic adjustments (128). Structured follow-up (encompassing pain intensity, functional status, side effects, and patient-reported outcomes) is critical to optimizing benefit-risk balance and sustaining functional gains over time (132).

#### 3.6.1 Key domains to assess

In mixed pain, comprehensive assessment extends beyond pain intensity, encompassing multiple biopsychosocial dimensions. Intensity is captured by validated tools such as the Numerical Rating Scale (NRS) and Visual Analog Scale (VAS), with proven responsiveness in chronic low back pain (133). Pain interference, reflecting daily activity limitations, is assessed with the Brief Pain Inventory (BPI), which correlates with disability outcomes (134).

Functional ability is evaluated using disease-specific tools like the Oswestry Disability Index (ODI) and Roland–Morris Disability Questionnaire, validated for chronic pain (133). Psychosocial factors, including catastrophizing, anxiety, and depression, are assessed with instruments such as the Pain Catastrophizing Scale and Hospital Anxiety and Depression Scale (HADS), key for identifying at-risk patients (135). Moreover, the Patient Global Impression of Change (PGIC) can be used as a patient-reported outcome measure that assesses the patient’s overall perception of improvement or deterioration in their condition since the beginning of treatment.

Health-related quality of life is measured using generic instruments like EQ-5D and SF-36. Incorporating patient-reported outcomes (PROs) enhances understanding of subjective treatment responses and complements clinician assessments (133). A multidimensional evaluation strategy (spanning intensity, interference, function, psychosocial status, quality of life, and PROs) aligns with best practices in mixed pain management.

#### 3.6.2 Monitoring tools

Effective monitoring employs various tools to capture the complex nature of mixed pain. Electronic pain diaries enable real-time tracking of fluctuations and responses, enhancing patient awareness and communication. Diary use improves pain intensity, mood, and function (136).

Electronic Health Records (EHRs) support systematic documentation of progress, adjustments, and care coordination, improving evaluation and treatment quality (137, 138). Wearable devices provide objective data on activity and sleep patterns, offering insight into pain-sleep relationships (139). Advanced wearables (e.g., polysomnography, AI-driven tools) enable high-resolution, longitudinal monitoring in real-world settings. Clinicians should inform patients thoroughly when applying wearable technologies (140).

Artificial intelligence may further enhance monitoring, though ethical considerations remain (141, 142). Collectively, these tools promote adaptive, patient-centered adjustments and proactive pain management.

#### 3.6.3 Red flags in monitoring

Vigilant monitoring identifies red flags signaling inadequate or unsafe therapy. One key concern is escalating opioid doses without functional improvement, warranting reassessment if exceeding ≥50 morphine milligram equivalents daily (143). Rising psychological distress (linked to worse outcomes on long-term opioids) also requires attention (144).

Functional decline despite treatment or signs of medication misuse (early refills, dose escalation, abnormal toxicology screens) indicate potential opioid use disorder and necessitate intervention (145).

Monitoring these domains with validated tools, clinical judgment, and prescription tracking ensures safe, effective mixed pain management and improved outcomes.

**Recommendation 14**

**Multidimensional assessment tools** We strongly recommend using validated multidimensional assessment tools (Brief Pain Inventory, Oswestry Disability Index, Pain Catastrophizing Scale) for comprehensive outcome monitoring (Strong recommendation, Low evidence). Moreover, we suggest implementing structured follow-up schedules with assessment intervals appropriate to treatment intensity and patient risk profile (Conditional recommendation, Low evidence).

### 3.7 Special populations

Mixed pain manifests differently across patient populations due to varying biological, psychosocial, and contextual factors, necessitating tailored therapeutic approaches. Psychosocial influences modulate pain perception and treatment responsiveness, underscoring the need for individualized care strategies (146).

#### 3.7.1 Elderly patients

Mixed pain in older adults commonly arises from musculoskeletal conditions (e.g., osteoarthritis) combined with neuropathic elements such as diabetic polyneuropathy. Approximately 40%–65% report musculoskeletal pain, and up to one-third experience neuropathic symptoms (147). Management must account for increased risks of adverse drug events and pharmacokinetic changes due to aging and polypharmacy. Guidelines recommend simplified regimens, lower dosages, and topical agents to minimize systemic risks (148). Cognitive impairment and frailty, which affect symptom reporting and treatment tolerability, must also be assessed routinely (149). Age-related pharmacokinetic and pharmacodynamic changes increase risks of adverse effects and drug–drug interactions. A “start low, go slow” principle is critical. In patients >75 years, topical formulations (lidocaine, capsaicin, NSAID patches) should be prioritized to minimize systemic exposure. Polypharmacy must be carefully reviewed to avoid drug interactions and adverse outcomes. Non-pharmacological therapies such as physiotherapy, occupational therapy, and balance training are essential to maintain function, prevent falls, and improve quality of life. Invasive procedures may be considered selectively but should take into account comorbidities, frailty, and anticoagulation status. A tailored, patient-centered model improves safety and outcomes in this vulnerable group.

#### 3.7.2 Pediatric patients

Mixed pain in children (arising from sickle cell crises, postoperative recovery, or oncology treatment) is frequently underrecognized. A 2023 systematic review in sickle cell disease showed that psychological interventions (e.g., cognitive-behavioral therapy, biofeedback) effectively reduce pain frequency and intensity, addressing neuropathic and nociceptive components (150). Inpatient therapies such as virtual reality and yoga, and outpatient approaches like massage, offer further benefit (151). Integrating these strategies into family-centered care models aligns interventions with patient and family needs, improving adherence and outcomes. Evidence on mixed pain in children is limited, and treatment should emphasize conservative and multimodal approaches. Pharmacological therapy must be guided by strict age- and weight-based dosing: paracetamol and NSAIDs may be used as first-line, while adjuvants (gabapentinoids, antidepressants) should only be prescribed under specialist supervision due to limited pediatric safety data. Non-pharmacological approaches—including cognitive-behavioral therapy, school reintegration support, and family-centered interventions—are particularly important, as children are highly vulnerable to psychosocial drivers of pain chronification. Interventional techniques should be reserved for highly selected cases and delivered in specialized centers.

#### 3.7.3 Cancer patients

Cancer-related mixed pain combines tumor-induced nociceptive mechanisms (e.g., bone compression) with treatment-related neuropathic processes (e.g., chemotherapy-induced neuropathy). Approximately 20% of cancer patients exhibit this mixed phenotype (152). The traditional WHO analgesic ladder often proves insufficient, with 39% experiencing inadequate pain relief; mechanism-based adaptations significantly improve outcomes (153). Current guidelines recommend integrating adjuvant agents (e.g., anticonvulsants, antidepressants) and early interventional techniques to better address complex cancer-related mixed pain (154). This mechanism-based, multimodal analgesic approach better aligns with the evolving phenotypic complexity of cancer-related mixed pain and optimizes patient outcomes within a personalized palliative care framework.

#### 3.7.4 Patients with psychiatric comorbidities

Psychiatric comorbidities (depression, anxiety, PTSD) amplify mixed pain through biopsychosocial pathways (155, 156). SNRIs and other psychotropic agents offer dual benefits for mood and pain symptoms (157). Integrated care involving pain specialists and mental health professionals optimizes pharmacological regimens, minimizes interaction risks, and addresses emotional drivers of pain, enhancing outcomes.

#### 3.7.5 Socioeconomically disadvantaged populations

Disadvantaged individuals face systemic barriers, including poor access to specialized care, limited health literacy, and higher rates of untreated chronic pain. Lower health literacy is linked to poor adherence and suboptimal outcomes (158), with disproportionate burdens of unmanaged pain reflecting social inequities (159). Culturally tailored education, mobile health solutions, and equity-focused programs are needed to promote engagement and improve long-term outcomes in these populations.

**Recommendation 15**

**Special population.** We strongly recommend adapting mixed pain treatment strategies to patient age, including simplified regimens and topical therapies for elderly patients, and family-centered, non-pharmacological approaches for pediatric patients (Strong recommendation, Very low evidence). We strongly recommend mechanism-based multimodal approaches for cancer patients with mixed pain (Strong recommendation, Low evidence). We strongly recommend close collaboration between pain specialists and mental health professionals in patients with mixed pain and psychiatric comorbidities (Strong recommendation, Moderate evidence). Future research should address the major knowledge gaps on mixed pain in minority and vulnerable populations, who remain underrepresented in clinical trials and face barriers to access and care. Tackling these disparities is crucial to ensure inclusive, equitable, and generalizable recommendations.

## 4 Ethical and practical considerations

Ethical principles are fundamental to the management of mixed pain. Its multifactorial nature requires not only clinical expertise but also a robust ethical framework to ensure patient-centered care. Central is the principle of autonomy, which mandates respect for patients’ rights to informed decision-making. In mixed pain, where treatment often involves trade-offs between efficacy, side effects, and personal values, shared decision-making is essential. Active patient engagement strengthens therapeutic alliances, enhances adherence, and respects individual goals (160).

The principle of beneficence obliges clinicians to optimize outcomes through evidence-based multimodal interventions, integrating pharmacological and non-pharmacological strategies. However, this must be balanced by non-maleficence, given the risks associated with opioids, anticonvulsants, and antidepressants, which may cause sedation, dependence, or systemic harm. Ethical care requires careful benefit-risk assessment and vigilant monitoring to prevent iatrogenic complications (161).

Justice demands equitable access to effective pain care. Yet disparities persist, with underserved populations facing barriers to multidisciplinary services. Addressing such inequities is an ethical imperative, requiring fair allocation of resources (including specialist care, rehabilitation, and psychosocial support) to ensure that all patients can benefit from integrated pain management (162).

Informed consent is central to ethical care, particularly in mixed pain where long-term pharmacotherapy, invasive interventions, or off-label treatments are common. Patients must be fully informed about their condition, treatment options, risks, and uncertainties. Compassionate communication and documented shared decision-making are essential, particularly when managing therapies with significant risks such as opioids or neuromodulation (163).

Resource limitations often constrain optimal multimodal care. In settings where comprehensive approaches or advanced modalities are unavailable, ethical management requires judicious resource allocation, telemedicine integration, and advocacy for system reform to enhance equity. Clinicians must deliver care within existing constraints while advocating for broader structural improvements (164).

Professional competence is equally vital. The complexity of mixed pain may exceed the expertise of individual providers. Recognizing limits and ensuring timely referral to specialized centers or m teams is ethically warranted. Collaborative care models are essential to deliver comprehensive, evidence-based treatment across medical, psychological, and rehabilitative domains (165).

Finally, increasing reliance on digital tools (EHRs, remote monitoring, telehealth) raises concerns regarding privacy and data security. In fact, ethical practice demands adherence to privacy regulations, transparency about data use, and robust cybersecurity. Informed consent for digital data use is essential to maintain trust and uphold ethical standards in technologically supported care (166). When integrating telemedicine, digital tools, and wearable monitoring into mixed pain management, adherence to established data privacy and security regulations is essential. In the European Union, the General Data Protection Regulation (GDPR) provides the legal framework for processing personal health data, mandating explicit patient consent, data minimization, and secure storage and transfer of sensitive information. In the United States, the Health Insurance Portability and Accountability Act (HIPAA) sets the standards for protecting patient health information, with specific requirements for confidentiality, integrity, and data access control. Other jurisdictions may apply additional regulations, such as the Personal Information Protection and Electronic Documents Act (PIPEDA) in Canada or comparable national laws. For clinical implementation, it is therefore crucial that institutions deploying digital health solutions ensure compliance with the relevant regional legal framework, establish clear protocols for data handling and storage, and provide transparent information to patients about how their data are collected, processed, and protected. This safeguards patient rights while fostering trust in the use of digital technologies for pain care.

In sum, ethical management of mixed pain requires more than clinical skill: it demands a steadfast commitment to autonomy, beneficence, non-maleficence, and justice, underpinned by transparency, resource stewardship, professional integrity, and data governance. These principles form the foundation of ethical, patient-centered pain care.

**Recommendation 16**

**Ethical and Equitable Mixed Pain Care**

We strongly recommend that mixed pain management be grounded in ethical principles, including comprehensive informed consent and shared decision-making that respect patient autonomy and ensure patients are fully informed of the complex nature of their condition, available treatment options, associated risks, and realistic outcomes. In parallel, healthcare systems should implement equitable policies to guarantee access to interdisciplinary care across geographic and socioeconomic barriers (Strong recommendation, Low evidence). Furthermore, we suggest establishing and maintaining professional competency standards through minimum qualifications and ongoing education for all providers involved in mixed pain management (Conditional recommendation, Low evidence).

## 5 Conclusion and future directions

Mixed pain presents a complex, multidimensional challenge in clinical practice, requiring a shift from traditional single-modality treatments to comprehensive, individualized strategies. These clinical recommendations provide a systematic framework for diagnosing mixed pain, integrating validated assessment tools with clinical reasoning to accurately characterize pain profiles. Pharmacological management should be mechanism-based, often necessitating multimodal combinations for effective relief. Concurrently, non-pharmacological interventions (including physical rehabilitation, psychological therapies, and integrative modalities) are essential for functional restoration and enhancing resilience. Integrated care models offer the most effective approach for sustained relief and improved quality of life, fostering coordinated efforts across clinical domains. Ethical responsibility, continuous outcome evaluation, and equitable access to care remain essential pillars of best practice. Table 2 summarizes key clinical recommendations for the diagnosis and management of mixed pain, organized by thematic chapters. Each recommendation aligns with a specific clinical domain and embodies a mechanism-based, patient-centered strategy.

**TABLE 2:** Summary of clinical recommendations for mixed pain management.

Recommendation	Topic	Key elements
1	Mechanism-based classification	Classify mixed pain based on relative contributions of nociceptive, neuropathic, and nociplastic components to guide targeted therapeutic approaches
2	Central sensitization assessment	Routine evaluation for clinical signs of central sensitization (hyperalgesia, allodynia, temporal summation) in patients with suspected mixed pain
3	Validated screening tools	Use validated screening tools (DN4, PainDETECT, LANSS) in combination with comprehensive clinical assessment to identify neuropathic components
4	Comprehensive clinical assessment	Include detailed pain history, physical examination with sensory testing, and systematic assessment of functional impact and psychosocial factors
5	Imaging and electrophysiology	Use advanced imaging (MRI) and electrophysiological studies (EMG/NCS) selectively based on clinical presentation rather than routinely
6	Red and yellow flag assessment	Systematic screening for red flags (serious pathology) and yellow flags (psychosocial risk factors) during initial evaluation
7	Multimodal pharmacotherapy	Use multimodal pharmacotherapy combining agents with different mechanisms of action rather than monotherapy
8	First-line combination therapy	Combine NSAIDs or acetaminophen with gabapentinoids (gabapentin/pregabalin) or SNRIs (duloxetine) as first-line therapy
9	Dual-mechanism opioids	Consider opioids with dual mechanisms (tramadol or tapentadol) over traditional opioids when opioid therapy is indicated
10	Topical agents	Prioritize topical agents (lidocaine patches, capsaicin) for localized mixed pain, particularly in elderly patients or those with multiple comorbidities
11	Multimodal non-pharmacological approach	Integrate multiple non-pharmacological interventions (physical therapy, psychological support, patient education) alongside pharmacological treatment
12	Early psychological intervention and education	Early integration of psychological interventions (CBT, ACT, MBSR) for patients with high pain-related distress; provide comprehensive patient education
13	Team composition and communication	Interdisciplinary teams should include minimum: physician, nurse, pharmacist, physiotherapist, psychologist; establish regular team meetings
14	Multidimensional assessment	Use validated multidimensional assessment tools (Brief Pain Inventory, Oswestry Disability Index, Pain Catastrophizing Scale); implement structured follow-up schedules
15	Age-specific and population-based adaptations	Modify treatment approaches based on patient age (simplified regimens for elderly, family-centered approaches for pediatric); mechanism-based multimodal approaches for cancer patients; close collaboration between pain specialists and mental health professionals for patients with psychiatric comorbidities
16	Ethical and equitable care	Ground management in ethical principles including comprehensive informed consent, shared decision-making, and equitable access policies; establish professional competency standards through minimum qualifications and ongoing education

Future research should focus on developing objective diagnostic tools (e.g., biomarkers, advanced imaging) and advancing system-level innovations through digital health and personalized medicine. Ultimately, managing mixed pain demands a holistic, person-centered approach that mobilizes all resources to alleviate suffering and restore function.
