# Supplementary material for: Mixed pain: clinical practice recommendations
Source: Front Med (Lausanne). 2025 Oct 9;12:1659490. doi: 10.3389/fmed.2025.1659490 (PMC12557516; doi:10.3389/fmed.2025.1659490)
Supplement: Supplementary file 1 [file Supplementary_file_1.docx]

**Supplementary Material**

The clinical practice recommendations presented in this document were developed through a consensus process involving systematic literature review combined with structured expert opinion collection. Given the limited availability of high-quality randomized controlled trials specifically addressing mixed pain as a distinct clinical entity, a modified Delphi methodology was employed to synthesize available evidence with expert clinical experience (1). A multidisciplinary expert panel was assembled comprising pain medicine specialists, neurologists, anesthesiologists, rehabilitation physicians, clinical pharmacologists, and patient care coordinators from the participating scientific societies. Panel members were selected based on predefined criteria including minimum five years of clinical experience in pain management, documented scholarly contributions through peer-reviewed publications, conference presentations, or educational activities related to mixed pain or multimodal analgesia, current membership in recognized professional pain management organizations and demonstrated commitment to evidence-based practice principles. The expert panel represented diverse geographic regions across three continents, including representatives from Fondazione Paolo Procacci (FPP), Federación Latinoamericana de Asociaciones para el Estudio del Dolor (FEDELAT), and African Society for Regional Anesthesia (AFSRA).

**Statement Development**

The scientific advisory committee conducted comprehensive literature analysis to identify critical domains requiring standardization and expert consensus. Key investigation areas were systematically categorized into six primary domains including the pathophysiology and mechanism identification, diagnostic approaches and assessment tools, pharmacological management strategies, non-pharmacological interventions, interdisciplinary care models and monitoring and outcome evaluation.

Statement formulation followed established guidelines for consensus development, utilizing the BRUSO framework (brief, relevant, unambiguous, specific, and objective) to ensure clarity and clinical applicability (2 ). An initial pool of 24 potential statements was developed through iterative review cycles by the scientific committee, ultimately refined to 16 carefully constructed statements addressing the most clinically relevant and evidence-informed aspects of mixed pain management.

**Delphi Survey Implementation**

The consensus evaluation was conducted using a secure, web-based platform ensuring participant anonymity and systematic data collection. Each expert received personalized access credentials and comprehensive instructions regarding evaluation procedures and response timelines. A 5-point Likert scale was implemented for statement assessment (1: strongly disagree; 2: disagree; 3: somewhat disagree; 4: agree; 5: strongly agree). This scale selection was based on established consensus methodology recommendations and successful application in previous delphi publications of our group (3,4). The consensus threshold was established a priori at ≥70% agreement, defined as the proportion of respondents selecting "agree" or "strongly agree" (scores ≥4 on the Likert scale). This threshold aligns with accepted standards in medical consensus research (5). Participants were provided with a two-week response period to complete the comprehensive evaluation, with systematic reminder communications sent at predetermined intervals to optimize response rates and ensure adequate time for thoughtful consideration of each statement.

**Statistical analysis**

Descriptive statistics were calculated for all participant demographic characteristics and statement responses. Agreement percentages were computed as the proportion of respondents scoring each statement ≥4 on the Likert scale. Response distribution patterns were analyzed to identify areas of strong consensus versus ongoing clinical uncertainty. All analyses were performed using R software v4.3.2 (R Foundation for Statistical Computing, Vienna, Austria, [www.r-project.org](http://www.r-project.org)).

**Supplementary Results**

The Delphi consensus process was completed with full participation from all 40 invited experts, achieving a 100% response rate. The expert panel demonstrated substantial professional diversity and extensive clinical experience in mixed pain management across multiple healthcare settings and geographic regions. The single-round Delphi process demonstrated remarkable success in achieving expert consensus across the investigated domains. All the statements successfully reached the predetermined consensus threshold of ≥70% agreement. Agreement levels across achieving statements ranged from 80% to 98%, with a median consensus level of 88%. Statements achieving the highest levels of expert agreement included those addressing fundamental safety protocols and documentation requirements. Specifically, Statement 16 regarding comprehensive documentation of treatment approaches achieved 98% agreement, Statement 3 addressing validated screening tools reached 95% agreement, Statement 11 concerning interdisciplinary team composition attained 95% agreement, and Statement 14 on multidimensional assessment tools achieved 95% agreement.

**Thematic analysis by clinical domain**

*Pathophysiology and Diagnostic Assessment (Statements 1-6)*

This foundational domain achieved consensus on all 6 statements, with agreement levels ranging from 80% to 95%. Experts demonstrated strong consensus regarding the integration of validated screening tools with comprehensive clinical assessment (Statement 3: 95% agreement), indicating universal recognition of evidence-based diagnostic approaches. The importance of systematic red flag and yellow flag assessment during initial evaluation received robust support (Statement 6: 92% agreement), reflecting widespread professional commitment to comprehensive risk stratification in mixed pain patients. Mechanism-based pain classification approaches (Statement 1: 88% agreement) and comprehensive clinical assessment (Statement 4: 88% agreement) also achieved strong consensus. Additionally, experts showed substantial agreement regarding imaging and electrophysiology utilization (Statement 5: 80% agreement) and mechanism-based classification principles (Statement 2: 90% agreement).

*Pharmacological Management Strategies (Statements 7-10)*

Pharmacological management recommendations achieved consensus across all 4 statements in this domain, with agreement levels ranging from 80% to 85%. The strongest consensus emerged around multimodal pharmacotherapy approaches (Statement 7: 85% agreement). Experts also demonstrated strong agreement regarding first-line combination therapy recommendations (Statement 8: 82% agreement) and prioritization of topical agents for localized mixed pain (Statement 10: 80% agreement). The endorsement of dual-mechanism opioids when opioid therapy is clinically indicated (Statement 9: 80% agreement) achieved consensus.

*Non-pharmacological Interventions and Interdisciplinary Care (Statements 11-14)*

This domain achieved consensus on all 4 statements, with particularly high agreement levels ranging from 85% to 95%. The strongest consensus emerged around interdisciplinary team composition requirements (Statement 11: 95% agreement) and multidimensional outcome assessment approaches (Statement 14: 95% agreement), indicating universal recognition that mixed pain management requires coordinated multidisciplinary expertise and evidence-based monitoring. Early psychological intervention integration (Statement 12: 90% agreement) and comprehensive patient education (Statement 13: 85% agreement) received robust endorsement, reflecting growing appreciation for the biopsychosocial nature of mixed pain.

*Special Populations and Ethical Considerations (Statements 15-16)*

The final domain addressing special population needs and ethical framework achieved consensus on both statements, with agreement levels of 82% and 98% respectively.

**Response Pattern Analysis**

The proportion of "strongly agree" responses (score 5) varied considerably across statements, ranging from 35% to 58% of total responses. This variation likely reflects different levels of confidence across the investigated topics, with higher "strongly agree" proportions observed for statements addressing well-established clinical practices compared to evolving or resource-dependent approaches. Subgroup analysis revealed no significant differences in consensus patterns based on geographic region, years of clinical experience, or institutional affiliation.

Detailed agreement rates for each of the 16 clinical practice statements developed through the Delphi consensus process for mixed pain recommendations are presented in Supplementary Figure 1.


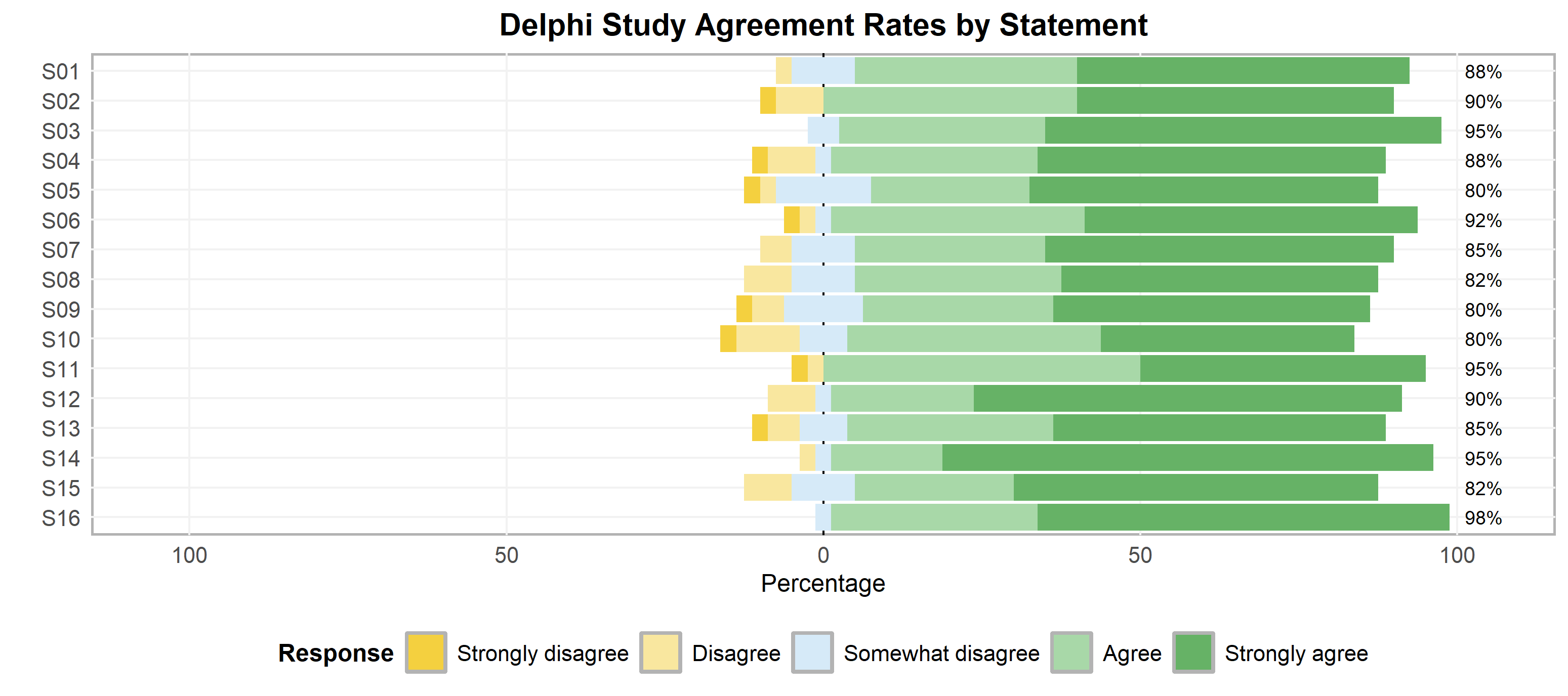


**Supplementary figure 1. Expert consensus agreement results for mixed pain clinical recommendations.** Distribution of expert agreement levels across 16 clinical practice statements for mixed pain diagnosis and management. Each horizontal bar represents the proportion of expert responses across a 5-point Likert scale (strongly disagree to strongly agree), with agreement percentages displayed on the right. All 16 statements (100%) achieved consensus, with agreement levels ranging from 80% to 98%. The highest consensus was achieved for comprehensive documentation requirements (S16: 98%), followed by validated screening tools (S03: 95%), interdisciplinary team composition (S11: 95%), and multidimensional assessment approaches (S14: 95%). The lowest consensus levels were observed for imaging and electrophysiology use (S05: 80%), dual-mechanism opioids (S09: 80%), and topical agents (S10: 80%), though all still exceeded the consensus threshold.

**Supplementary References**

1. Nasa P, Jain R, Juneja D. Delphi methodology in healthcare research: How to decide its appropriateness. World J Methodol. 2021;11:116–29.

2. Peterson R. Constructing Effective Questionnaires [Internet]. 2455 Teller Road, Thousand Oaks California 91320 United States: SAGE Publications, Inc.; 2000 [cited 2022 Dec 4]. Available from: https://methods.sagepub.com/book/constructing-effective-questionnaires

3. Leoni MLG, Occhigrossi F, Tenti M, Raffaeli W, ISAL Research Study Group. Endoscopic Epidurolysis for the Management of Chronic Spinal Pain: A Delphi-Based Italian Experts Consensus. Pain Ther. 2025;14:339–57.

4. Occhigrossi F, Carpenedo R, Leoni MLG, Varrassi G, Chinè E, Cascella M, et al. Delphi-Based Expert Consensus Statements for the Management of Percutaneous Radiofrequency Neurotomy in the Treatment of Lumbar Facet Joint Syndrome. Pain Ther. 2023;12:863–77.

5. Trevelyan EG, Robinson PN. Delphi methodology in health research: how to do it? European Journal of Integrative Medicine. 2015;7:423–8.
